# *In Situ* Assembly of 3-(Tetrazol-5-yl)triazole Complexes with Ammonium Perchlorate for High-Performance Energetic Composites

**DOI:** 10.1021/acsami.4c20164

**Published:** 2025-01-10

**Authors:** Ke-Juan Meng, Kunyu Xiong, Iftikhar Hussain, Momang Tian, Xinwen Ma, Yuxiang Li, Qi-Long Yan, Kaili Zhang

**Affiliations:** † Department of Mechanical Engineering, City University of Hong Kong, 83 Tat Chee Avenue, Hong Kong, SAR, China; ‡ State Key Laboratory on Solid Rocket Propulsion, Northwestern Polytechnical University, Xi’an 710072, China

**Keywords:** energetic composites, energetic coordination polymers, high reactivity, combustion performance, pressure output

## Abstract

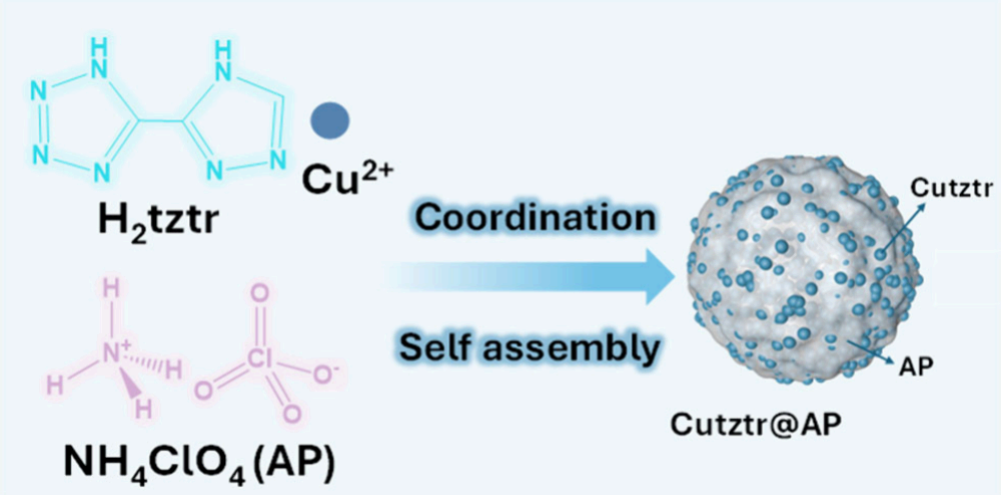

Advanced energetic composites possess promising properties and wide-ranging applications in explosives and propellants. Nonetheless, most metal-based energetic composites present significant challenges due to surface oxidation and low-pressure output. This study introduces a facile *in situ* method to develop energetic composites Cutztr@AP through the intermolecular assembly of nitrogen-rich energetic coordination polymers and high-energy oxidant ammonium perchlorate (AP). Morphological analysis reveals the unique structure of Cutztr@AP, where Cutztr is distributed throughout the interior and surface of the AP particles. The nonisothermal thermodynamic analysis reveals a heat release of 2378.2 J g^–1^ for Cutztr@AP_2_, outperforming the Cutztr/AP_2_ achieved through ultrasonic mixing (2000 J g^–1^). Notably, Cutztr@AP_2_ exhibits promising combustion and pressure output performances, including a significantly shorter duration, a larger flame area, and higher pressure values. This novel and facile preparation technique and microstructure design approach holds significant promise for high-performance propellants, gas generators, and other related applications.

## 1 Introduction

High-energy composites refer to high-energy powders that combine fuel and oxidizer particles, releasing heat and gas during decomposition and combustion. These materials can be widely used in propellants, gas generators, explosives, and pyrotechnics.^1^ Furthermore, they can integrate the advantages of individual energetic materials such as the incorporation of high-energy oxidants that enhance the oxygen balance and reduce the mass-transfer distance.^2^ For instance, nanoaluminum/nitrocellulose composite particles with a shorter distance between the fuel and oxidizer enhance the propellant burning rates and reduce particle agglomeration on the propellant surface.^3^ The energy level and active component content in energetic composites are fundamental factors influencing the overall energy level, while factors like the interfacial contact area between the fuel and oxidizer and mixing uniformity impact the energy release rate. Energetic composites can achieve tailored energetic properties for various applications by optimization of these parameters.

Metal fuels such as aluminum (Al)^4^ and boron (B)^5^ are favored in energetic composites due to their high combustion enthalpy, availability, and stability in high-energy applications.^6^ However, these fuels face significant challenges due to surface oxidation and a lack of gas-producing elements like C, H, O, and N, leading to issues like low gas production, incomplete reactions, and high ignition temperatures.^7−9^ Efficient heat transfer requires a large amount of gas products and high pressure, but metal-based energetic composites often fall short of generating sufficient gas, limiting their application.

To address these limitations, the synthesis of energetic coordination polymers (ECPs) by linking metal ions with nitrogen-rich ligands has gained attraction.^10−13^ This approach has shown promise in creating novel high-performance energetic materials due to the involvement of nitrogen-rich ligands such as tetrazole, triazole, furazan, tetrazine, and triazine.^14−20^ Among these ligands, triazole and tetrazole groups are particularly noteworthy for their metal-bridging capabilities and stability-enhancing properties, with 3-(tetrazol-5-yl)triazole (H_2_tztr) standing out for its 70% nitrogen content and versatile functionalities.^21,22^ When ECPs are combined with ammonium perchlorate (AP), a widely studied oxidizer known for its high oxygen content and strong oxidizing properties, the oxygen balance of energetic composites can be improved, potentially enhancing the reaction efficiency significantly.^23−25^ Therefore, this strategic integration of ECPs and AP holds promise for advancing the performance and applicability of energetic composites in various energetic materials applications.

To enhance the reaction rate of energetic composites, a crucial aspect involves ensuring intimate mixing between the oxidizers and fuels. Various preparation methods have been developed. Among them, ultrasonic mixing is the simplest and most commonly used method for energetic composites preparation and generates heat that can be problematic for sensitive energetic material.^26^ Electrospray encapsulates fuel into the oxidizer, enhancing interactions between the fuel and oxidizer and resolving aggregation issues. However, it requires specialized equipment and solvents, leading to increased operational costs.^27−29^ The sol–gel process precisely controls the composition, density, morphology, and particle size of target materials at the nanoscale. However, it is time-consuming and operationally complex.^30−32^ Mechanical ball milling significantly reduces the reaction activation energy, refines grains, and improves the powder activity. However, they may produce a wide particle size distribution. Recently, Liu et al. designed intermolecular energetic materials based on AP and 5-aminotetrazole through a coordination-driven *in situ* self-assembly method.^33^ This strategy effectively boosted the combustion rate of the composite material by reducing mass-transfer distances between the fuel and oxidizer, thereby enhancing the energy release rate and combustion efficiency significantly.

Herein, Cutztr@AP is synthesized using an *in situ* assembly approach, demonstrating superior reactivity, enhanced combustion performance, and increased pressure output compared to Cutztr. In addition, Cutztr/AP is synthesized via ultrasonic mixing to study the impact of morphology on the performance. The morphology and composition of the prepared Cutztr and Cutztr@AP are analyzed using techniques like scanning electron microscopy, X-ray diffraction, and X-ray photoelectron spectroscopy. Thermal reactivity is evaluated using simultaneous thermogravimetry–differential scanning calorimetry, while combustion properties and pressure outputs are assessed through open-burning and closed-bomb experiments. These comprehensive analyses provide valuable insights into the developed energetic composites.

## 2 Experimental Section

3-(1*H*-Tetrazol-5-yl)-1*H*-triazole (H_2_tztr) and prepared energetic coordination polymers (ECPs) are hazardous materials, explosions of which may occur under certain conditions. Although we had no problems during the experiments and in handling the complexes and composites, appropriate safety precautions such as the use of safety glasses, face shields, and plastic spatulas should be taken, especially when the compounds are prepared on a large scale.

### 2.1 Raw Materials

Copper nitrate trihydrate [Cu(NO_3_)_2_·3H_2_O; purity >99.5%] was purchased from Shanghai Aladdin Biochemical Technology Co., Ltd. H_2_tztr used in the experiment was purchased from Jinan Henghua Sci. & Tec. Co., Ltd. Deionized water was obtained from a Milli-Q+ purification system (18 MΩ·cm). Ammonium perchlorate (AP) was obtained from China Academy of Engineering Physics. ACS-grade concentrated ethanol absolute was purchased from Anaqua Global International Inc., Ltd. (Hong Kong). All reagents were used without further purification.

### 2.2 Preparation of Cutztr, Cutztr/AP, and Cutztr@AP

The divalent transition-metal complexes were prepared by combining solutions of H_2_tztr and copper(II) salts at a molar ratio of 1:1 by a coordination reaction. To obtain the target ECPs, 137 mg (1 mmol) of H_2_tztr was dissolved in a mixed solution of 55.2 mL of deionized water and 13.8 mL of absolute ethanol, and the solution was magnetically stirred at 60 °C for 1 h until fully dissolved. Afterward, Cu(NO_3_)_2_·3H_2_O (242 mg, 1 mmol) was added to the above solution to react for 1 h with stirring. Then, the reaction mixture was heated at 60 °C for 24 h, followed by cooling naturally to room temperature. Finally, a blue powdered product (Cutztr) was obtained after centrifuging, washing, and drying, with a yield of approximately 79%.

The composites of Cutztr and AP mixed in mass ratios of 1:1, 1:2 and 1:3 were named Cutztr/AP_1_, CutztrAP_2_ and Cutztr/AP_3_, respectively. Taking Cutztr/AP_1_ as an example, 100 mg of Cutztr and 100 mg of AP were added to the test tube; then approximately 1 mL of ethanol absolute was added to the tube, and the mixtures were ultrasonicated for 4 h to ensure thorough mixing. After that, the samples were placed in an oven at 60 °C for 12 h to remove any remaining ethanol absolute. The powder was very easily ground with a mortar and pestle until the consistency of each sample was that of a loose powder.

Cutztr@AP was synthesized using an *in situ* assembly approach. The feed ratio of the raw materials was the same as that of Cutztr/AP, and the prepared composites were named Cutztr@AP_1_, Cutztr@AP_2_, and Cutztr@AP_3_, respectively. Taking Cutztr@AP_1_ as an example, the specific preparation process consisted of 137 mg of H_2_tztr being completely dissolved in a mixed solution of deionized water and absolute ethanol with magnetic stirring at 60 °C for 1 h. Afterward, 300 mg of AP was added to the obtained solution at room temperature, and a homogeneously dispersed solution was obtained after magnetic stirring for 1 h. Subsequently, 242 mg (1 mmol) of Cu(NO_3_)_2_·3H_2_O was slowly added to the above mixture, and a sky-blue solution was obtained after stirring and reaction for 1 h. The solution was transferred to the oven, and sky-blue precipitation was obtained after reaction at 60 °C for 24 h. Finally, Cutztr@AP_1_ was obtained after centrifuging, washing, and drying.

### 2.3 Morphological and Compositional Characterization

The morphology of the Cutztr and Cutztr@AP energetic composites was studied by field-emission scanning electron microscopy (SEM; FEI Quanta 450) coupled with energy-dispersive spectroscopy (EDS). The composition was determined through X-ray diffraction (XRD; Rigaku SmartLab) at 30 kV by using Cu Kα radiation (λ = 1.5418 Å). X-ray photoelectron spectroscopy (XPS; Escalab 250Xi) was performed using Al Ka excitation radiation under vacuum lower than 10^–7^ Pa.

### 2.4 Thermal Behaviors

The thermal decomposition behavior of Cutztr and Cutztr-based composites was studied by using thermogravimetry and differential scanning calorimetry (TG–DSC; Mettler TGA/DSC 3+). The heating rate was 10 °C·min^–1^ in the temperature range of 50–600 °C. After that, the derivative of thermal gravimetry (DTG) was obtained by calculating the first-order differential for temperature. A minimum of three measurements were taken for each composite under identical experimental conditions to quantify the error.

### 2.5 Open-Burning Experiments

An open-burning experiment of Cutztr and energetic composites was conducted by homemade equipment. Typically, a 7 mg loose sample was loaded into an alumina crucible (6 mm in inner diameter and 4 mm in depth). It was ignited by a nichrome ignition wire (0.25 mm in diameter and 10 cm in length) in an air atmosphere at a current of 2.7 A. The combustion was recorded by a high-speed camera (Phantom VEO 710) with a sampling rate of 10000 frames·s^–1^, and the distance between the camera and sample during the combustion process was around 70 cm.

### 2.6 Closed-Bomb Experiments

A closed-bomb experiment was conducted to test the dynamic pressure curve within a chamber during the combustion reaction of Cutztr and energetic composites. Specifically, a 20 mg loose sample was placed in a confined cell with a fixed volume of 8 mL and ignited by a nichrome wire (0.2 mm in diameter and 5 cm in length). The dynamic pressure during the reaction process was measured by a piezoelectric pressure sensor (PCB Piezotronics, Model 112B05) attached to the confined cell. The pressure signal was converted into a voltage signal through a sensor signal conditioner (PCB Piezotronics, Model 482C54) and subsequently recorded on an oscilloscope.

## 3 Results and Discussion

### 3.1 Morphological and Structural Analysis

**Figure 1:**
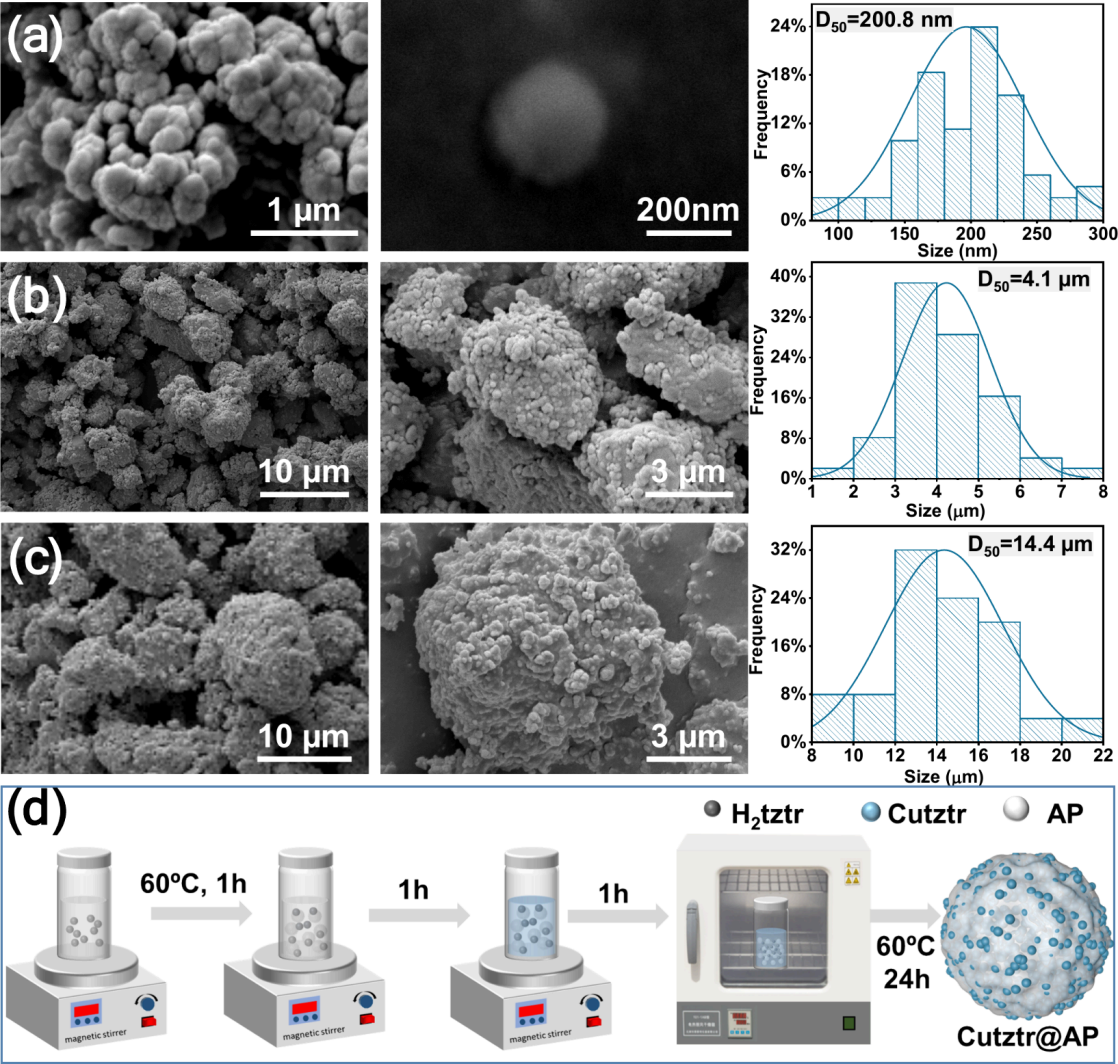
SEM results of (a) Cutztr, (b) Cutztr/AP_2_, and (c) Cutztr@AP_2_ and (d) the preparation process of Cutztr@AP.

The SEM image (Figure 1a) revealed that Cutztr spheroidal particles ranged in diameter from 150 to 200 nm. According to the EDS results (Figure S1), the atomic ratio of the elements Cu to N is approximately 1:7. Combined with the TG curve, it has no weight loss until 250 °C. It is presumed that no water of crystallization is involved in the coordination, and, therefore, its possible chemical formula is CuC_3_N_7_. Subsequent ultrasonic mixing with AP in ethanol at a 1:2 mass ratio resulted in the formation of multiple loosely stacked Cutztr/AP_2_ irregular microparticles with size of 3–6 μm, as depicted in Figure 1b. Notably, the SEM images show a nearly uniform distribution of Cutztr nanoparticles on the surface of AP. The SEM image of raw AP is given in Figure S2. Furthermore, SEM images of Cutztr/AP_1_ and Cutztr/AP_3_ with varying AP content have been displayed in Figure S3. Despite changes in the AP mass, a consistent appearance of uniformly dispersed nanoparticles suggested an effective distribution of Cutztr on AP surfaces. Conversely, when the *in situ* assembly approach was employed to create Cutztr@AP_2_, a distinct morphology emerged compared to Cutztr/AP_2_, as illustrated in Figure 1c. This new formation indicated that Cutztr not only exists on the AP surface but also is coated inside AP particles, resulting in larger particles exceeding 10 μm in size, supported by particle size distribution analysis. Therefore, the close contact between Cutztr and AP is likely to lead to a strong mutual interaction between the components. Similarly, Cutztr@AP_1_ and Cutztr@AP_3_ showed the same structure (Figure S4) as Cutztr@AP_2_, confirming the consistency and reliability of this preparation method. The EDS results of Cutztr@AP_1_ further demonstrate the uniform distribution of AP (Figure S5). The contact between Cutztr and AP is schematically illustrated in Figure 1d.

**Figure 2:**
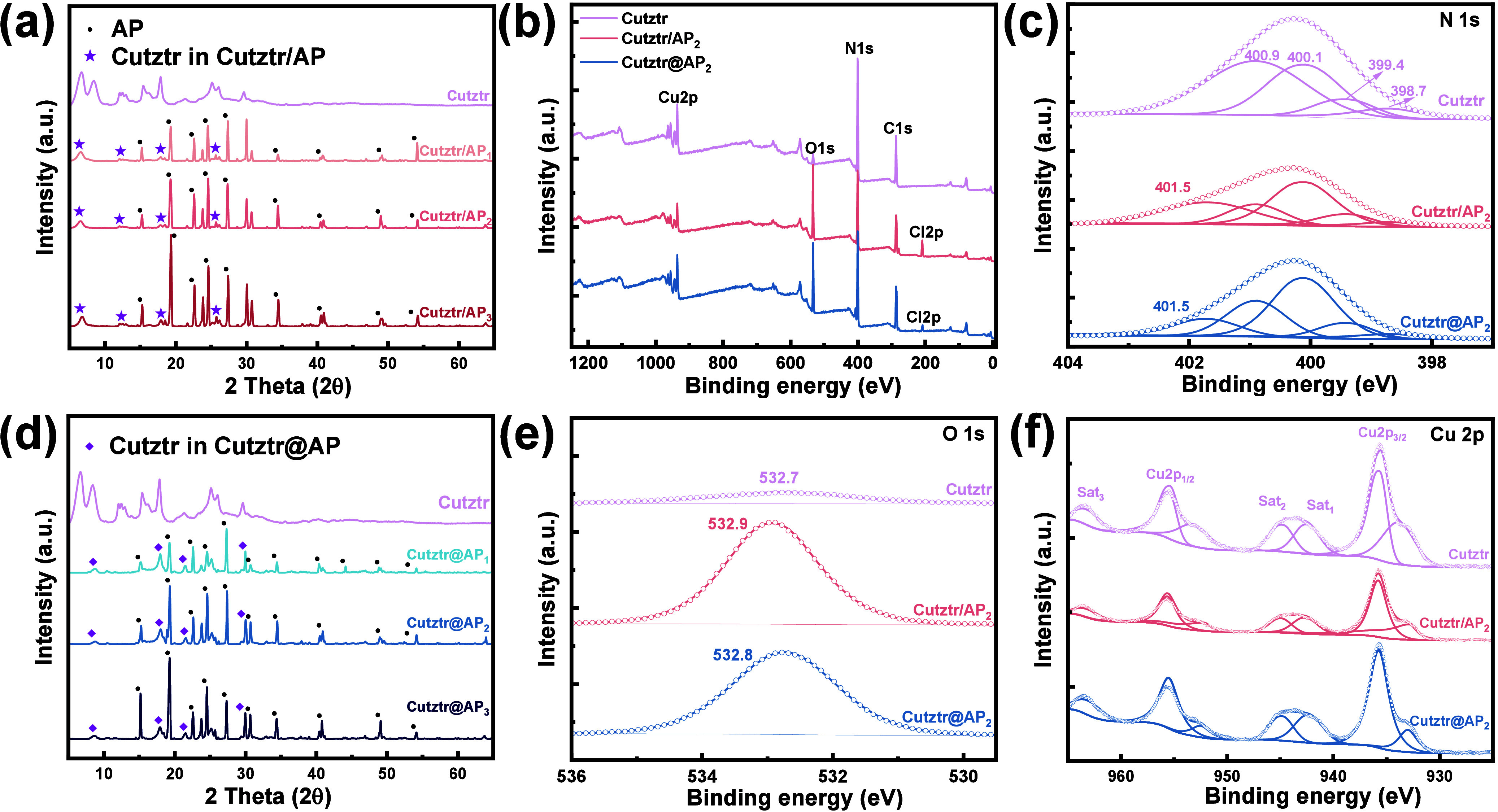
(a) XRD patterns of Cutztr and Cutztr/AP. (d) XRD patterns of Cutztr and Cutztr@AP. (b) Survey spectra of Cutztr, Cutztr/AP_2_, and Cutztr@AP_2_. High-resolution XPS spectra and deconvolution results of N 1s (c), O 1s (e), and Cu 2p (f).

XRD analysis was conducted to verify the structures of the prepared energetic materials, as shown in Figure 2a,d. Several diffraction peaks were observed from Cutztr, which were located at 6.5°, 8.5°, 12.5°, 15.4°, 17.8°, and 25.2°. These peaks did not align with those of known materials, confirming the novel nature of the synthesized materials. Recently, several ECPs based on H_2_tztr and Cu^2+^ have been synthesized,^21,22,34^ while the XRD peaks are not consistent with Cutztr in this work, which could be attributed to the three reversible types of protonated and deprotonated modes and six possible coordination modes.^35^ It could be found that the difference of Cutztr in ultrasonic mixed Cutztr/AP is comparatively small, proving that the ultrasonic mixing process changes the structure of Cutztr slightly. The diffraction peaks of Cutztr at 8.5° do not appear in all Cutztr/AP samples because of interaction between Cutztr and AP during the ultrasonic process. Nevertheless, most of the diffraction peaks of AP (PDF 43-0648) are maintained in all Cutztr/AP samples, and the intensity of these diffraction peaks increases as the AP content increases. On the contrary, the XRD patterns of Cutztr@AP samples show great changes. There is no diffraction peak at 6.5 and 12.5° for Cutztr, indicating that the growth of these planes is inhibited under a one-pot approach. However, the relative intensity of diffraction peaks at 17.9 and 25.2° increases significantly compared to that of Cutztr/AP, demonstrating that the Cutztr crystals in Cutztr@AP mainly grow along these planes. Likewise, all diffraction peaks of AP are maintained in all Cutztr@AP samples, and the relative intensity increases as the AP content increases.

XPS measurements were used to study the surface elemental composition and chemical properties of samples, verifying the effect of AP on the crystal structures of Cuzttr/AP and Cutztr@AP. In Figure 2b, the survey spectrum of Cutztr reveals the presence of Cu, N, C, and O. The weak O 1s signal can be attributed to absorbed oxygen or water.^36,37^ Conversely, as shown in Figure 2e, the intensity of O 1s of Cuzttr/AP and Cutztr@AP is obviously higher than that of Cutztr, which could be indexed to O–Cl bonds in AP.^38^ Moreover, the lower O–Cl binding energy in Cutztr@AP compared to Cutztr/AP suggests a reduction in the electron density of the O–Cl bond due to orbital interactions between Cutztr and AP, indicating a tighter binding of Cutztr and AP in Cutztr@AP. The appearance of the Cl 2p peak at around 210.0 eV in both Cutztr/AP and Cutztr@AP is attributed to Cl–O bonds in AP.^39^

The N 1s spectrum (Figure 2c) of Cutztr was deconvoluted into four peaks at different binding energies of 399.4, 398.7, 400.1, and 400.9°, corresponding to the stretching vibration of the linkage of Cutztr (Cu–N bond) and tetrazole–triazole including N=N—N, N—N=N, and N—N=C, respectively.^40^ After the AP combination forms a complex, it is reasonable to expect that the N–H functional group appearing at 401.5 eV belongs to AP.^41^

Additionally, the Cu 2p spectra (Figure 2f) of all samples exhibit characteristics of Cu^2+^ complexes with distinct satellite peaks alongside the main Cu 2p_3/2_ and Cu 2p_1/2_ peaks.^42^ The Cu 2p_3/2_ spectrum was fitted with two peaks at 933.2 and 935.7 eV, suggesting two chemical states of Cu in Cutztr. The peak located at 933.2 eV is assigned to Cu^2+^ because its binding energy is close to the value in ref (43). Another binding energy of Cu 2p_3/2_ (*E*_b_ = 935.7 eV) in all samples is very close to that of Cu 2p_3/2_ (*E*_b_ = 935.5 eV), suggesting that the chemical valence of Cu at 935.7 eV is also bivalent and its chemical environment is similar to that of copper phthalocyanine.^44^ The C 1s spectrum, as shown in Figure S6, exhibits two peaks located at 284.8 and 286.8 eV, which could be assigned to the C–C and C–N present in the ligands.^45^

### 3.2 Thermal Analysis

**Figure 3:**
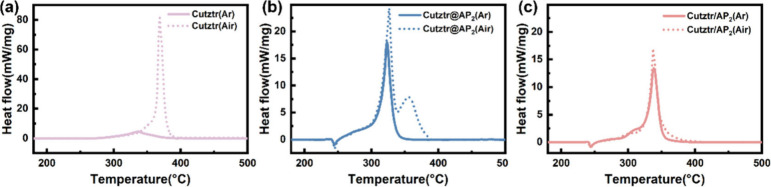
DSC curves of (a) Cutztr, (b) Cutztr@AP_2_, and (c) Cutztr/AP_2_ under Ar and air atmospheres.

The thermal behavior of the as-prepared samples was studied using a TG–DSC instrument. The samples were measured in covered 70 μL Al pans at a heating rate of 10 °C·min^–1^ (sample mass: 1 ± 0.1 mg). The heat flow and mass loss curves were recorded in the temperature range of 50–500 °C, and the detailed data are provided in Table S1. The heat release was obtained by integrating the shadow area on the DSC curves. The DSC curves depicted in Figure 3 illustrate the thermal behavior of Cutztr, Cutztr/AP_2_, and Cutztr@AP_2_ under both Ar and air atmospheres. It is evident that the maximum heat flow (∼80 mW·mg^–1^) and heat release (6460 J·g^–1^) of Cutztr in an air atmosphere are significantly higher than those in an Ar atmosphere (∼6 mW·m^–1^ and 1018 J·g^–1^), which indicates that Cutztr is negatively oxygen balanced. It is likely that, during thermal decomposition in an air atmosphere, Cutztr undergoes a chemical reaction with oxygen, thereby promoting energy release. In contrast, when Cutztr and AP are combined into composite materials, the difference between the maximum heat flow and heat release in Ar and air is significantly reduced, which further proves that Cutztr is a negative oxygen material and the addition of AP improves its oxygen balance.

**Figure 4:**
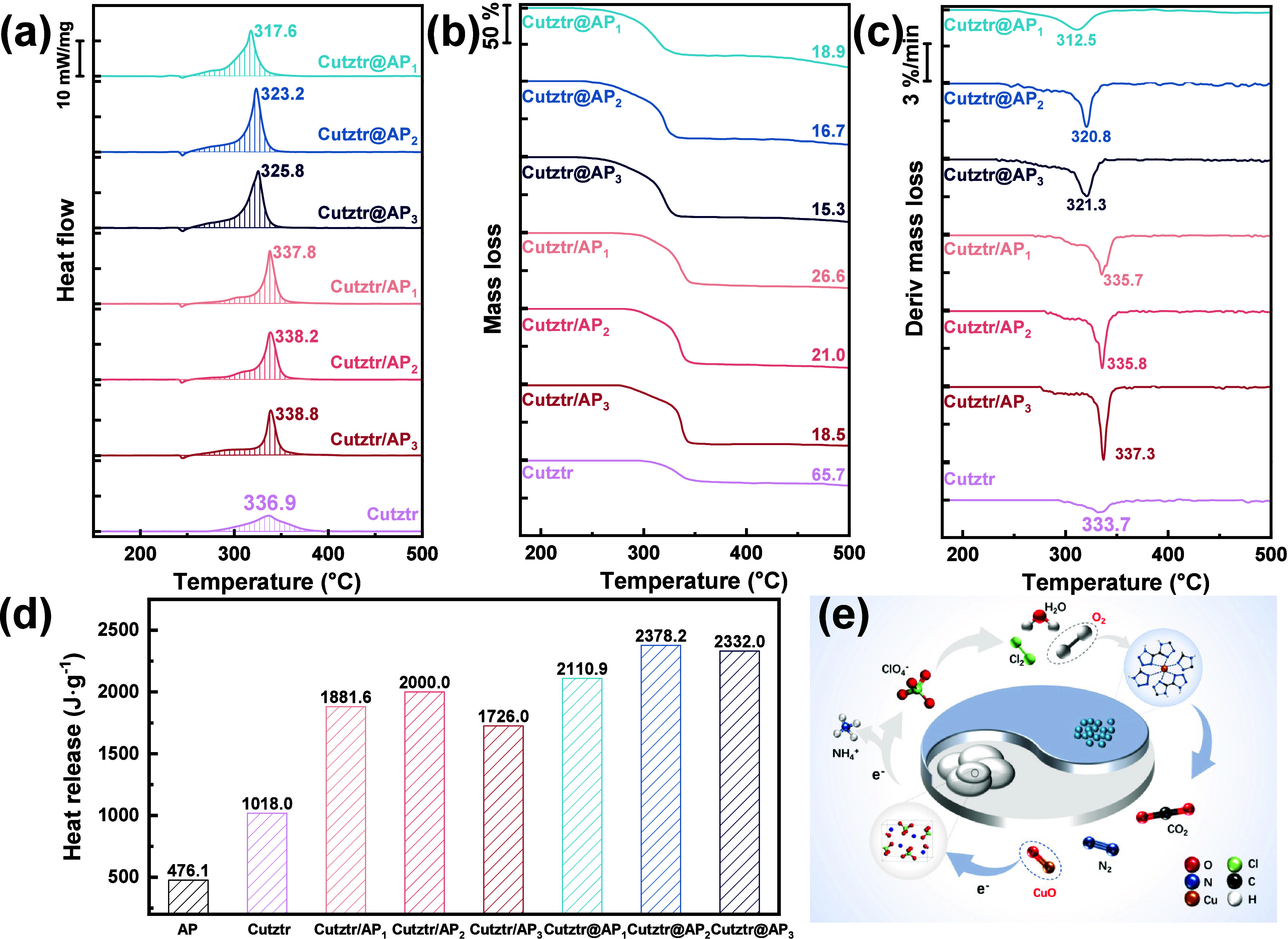
(a) DSC, (b) TG, and (c) DTG curves of Cutztr, Cutztr/AP, and Cutztr@AP under an Ar atmosphere. (d) Heat release of AP, Cutztr, Cutztr/AP, and Cutztr@AP. (e) Schematic illustration of the reaction between Cutztr and AP.

Figure 4 shows the DSC, TG, and DTG curves of Cutztr, Cutztr/AP, and Cutztr@AP under an Ar atmosphere. As shown in Figure 4b, the Cutztr only experienced a gradual weight loss of about 34% from 280 to 360 °C, which was attributed to the breakage of C–N and N–N bonds in the H_2_tztr ligand, leading to collapse of the Cutztr structure. This process corresponds to the exothermic stage of Cutztr in the DSC curve, indicating the release of gaseous products such as N_2_.^46^ When the TG-DSC curves of AP (Figure S7) and Cutztr were compared, notable differences were observed in the thermal behaviors of Cutztr/AP and Cutztr@AP. The decomposition of AP typically involves an initial endothermic stage followed by two exothermic stages. The endothermic stage corresponds to the transition of AP from an orthorhombic to a cubic crystal structure at 247.2 °C with no mass loss. This is followed by the low-temperature decomposition (LTD) stage between 290 and 350 °C, predominantly producing gaseous HClO_4_ and NH_3_.^25^ Subsequently, the high-temperature decomposition (HTD) stage at 370–460 °C involves further reactions producing gas mixtures like N_2_O, NO, O_2_, and HCl, releasing a large amount of heat and energy.^47^

After Cutztr is combined with AP, thermal decomposition of both Cutztr/AP and Cutztr@AP experiences an endothermic and a wide exothermic stage, which is likely due to the overlapping decomposition of Cutztr and the LTD of AP. This interaction promotes energy release and complete reaction. Additionally, CuO, an intermediate product of this reaction and a typical semiconductor material, accelerates electron transfer on the surface of AP, providing abundant electron-transfer pathways in the redox reaction cycle, thereby improving the reactivity of energetic composites, as shown in Figure 4e.^9,48^ It is worth noting that the exothermic peak temperatures of Cutztr/AP and Cutztr@AP show different trends. As illustrated in Figure 4a, the exothermic peak temperature of Cutztr/AP is approximately 338 °C, slightly higher than that of Cutztr (336.9 °C). In contrast, the exothermic peak temperature of Cutztr@AP is around 320 °C, which is lower than that of Cutztr. This suggests that a shorter distance between the oxidizer and fuel accelerates the mass-transfer rate.

For Cutztr/AP, the endothermic peak at about 245 °C corresponds to the crystal transformation of AP, and the subsequent exothermic process is in the range of 248–370 °C. As the AP content in Cutztr/AP increases, the exothermic peak temperature gradually increases and the residual mass after the reaction gradually decreases, indicating a more complete decomposition. Similarly, Cutztr@AP also exhibits an endothermic peak at about 245 °C, associated with Cutztr/AP. However, the subsequent exothermic process occurs in the range of 248–350 °C, with the peak width decreasing by approximately 20 °C compared to Cutztr/AP, leading to a further reduction in the residual mass. The exothermic and DTG peak temperatures increase progressively with the higher AP content, while the residual mass decreases. Additionally, the heat release of energetic materials is a key parameter, reflecting the energy level. The heat release of Cutztr@AP_2_ is about 2378 J·g^–1^, higher than that of Cutztr/AP (1726–2000 J·g^–1^), and the independent heat releases from Cutztr_2_ and AP are combined.

### 3.3 Combustion Performance

**Figure 5:**
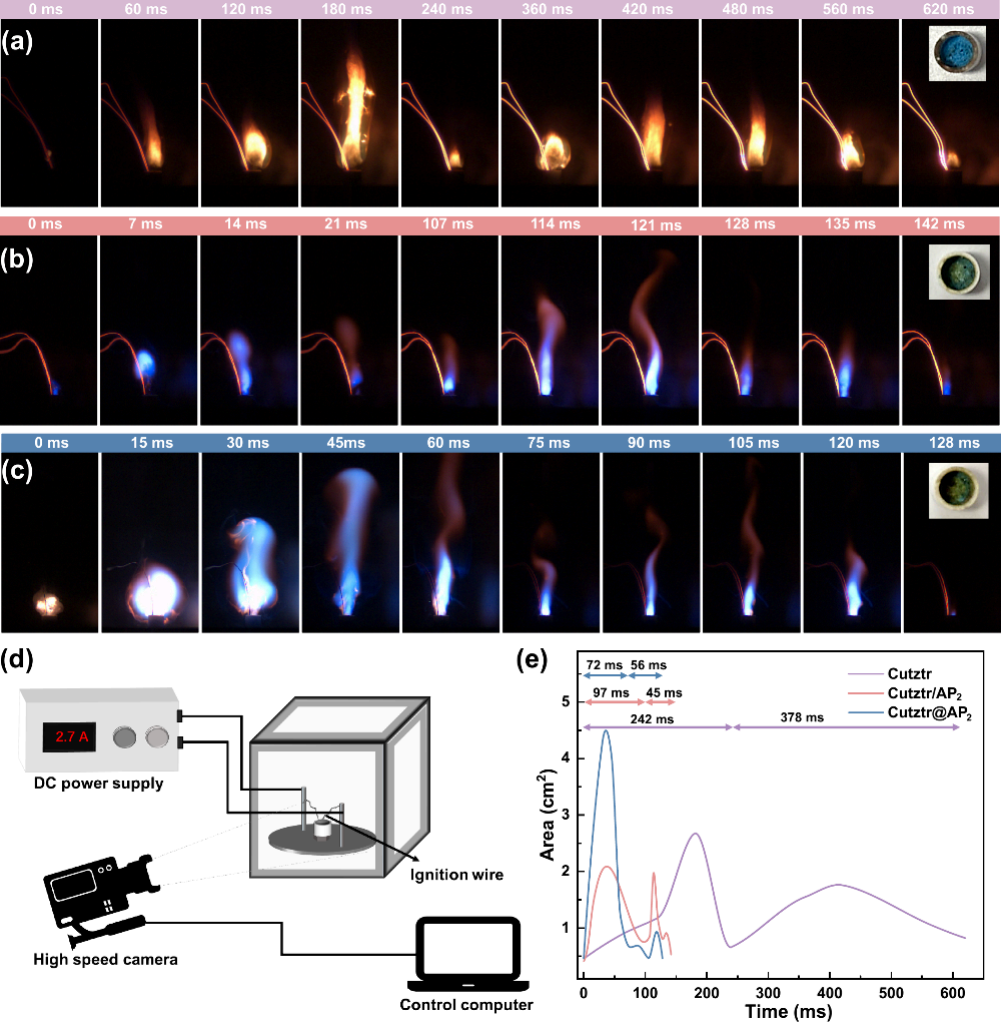
Sequential images of the combustion results of (a) Cutztr, (b) Cutztr/AP_2_, and (c) Cutztr@AP_2_. (d) Open-burning test equipment. (e) Variation of the flame area with time obtained by the FCM algorithm.

Open-burning experiments of Cutztr, Cutztr/AP, and Cutztr@AP are conducted in an air atmosphere using the homemade equipment as shown in Figure 5d. The combustion sequential images of ∼7 mg of loose powder samples loaded into alumina crucibles captured by a high-speed camera with the same exposure time and frame rate are shown in Figure 5a–c. To calculate the area of flame during the combustion process, the unsupervised machine-learning Fuzzy C-means (FCM) algorithm is used to segment the flame region and the background of the combustion. The typical segmentation results of Cutztr, Cutztr/AP, and Cutztr@AP are shown in Figure S8. The FCM algorithm, based on fuzzy theory, maximizes the similarity of data objects within a class, while minimizing the similarity between classes.^49^ The details of this algorithm are shown in the Supporting Information, and the calculated flame area variation curve with time is shown in Figure 5e.

It was observed that Cutztr exhibited a relatively slow reaction rate and long burning duration (more than 600 ms), attributed to the oxygen deficiency, leading to inadequate slow combustion. In contrast, both Cutztr/AP_2_ and Cutztr@AP_2_ exhibited shorter reaction time (around one-fifth of that of Cutztr), indicating faster energy release facilitated by the addition of AP. Meanwhile, Cutztr@AP_2_ exhibited a faster energy release rate and a larger flame area (about 4.5 cm^2^), suggesting better integration of Cutztr and AP within the structure, reducing the separation between the fuel and oxidizer, and promoting a faster and more complete reaction. As shown in the inset of Figure 5, the unreacted blue Cutztr sample remains in the alumina crucible, while only part of the green copper chloride combustion product remains after the combustion of Cutztr/AP_2_ and Cutztr@AP_2_, confirming that AP participates in the combustion reaction as an oxidizing agent. High-speed photography and flame area calculation results indicated that all samples underwent two stages of combustion, possibly due to the reaction process, leading to rapid dissipation of hot spots and energy and impacting sustained combustion. Notably, the first stage of Cutztr/AP_2_ and Cutztr@AP_2_ was longer than the second stage, while the reverse was true of Cutztr, further demonstrating that the addition of AP could improve the rate of energy release. Additionally, the combustion of Cutztr evinces a conspicuous yellow flame, while Cutztr/AP_2_ and Cutztr@AP_2_ exhibit bright-blue flames, in which Cutztr@AP_2_ shows incandescence followed by a conspicuous blue flame, accompanied by a significant amount of gas in the surroundings. It is well-known that flame colors are generated through flame reaction. The flame colors are a result of flame reactions, with chlorine sources producing light-emitting species, such as CuCl_2_, efficient emitters in the visible spectrum. Chlorine easily combines with metals to produce molecular radiation and emit bright characteristic flames.^50^ Therefore, the combustion product of Cutztr may be CuO; Cutztr/AP_2_ and Cutztr@AP_2_ likely follow a reaction resulting in CuCl_2_ formation (eq 1).
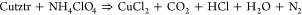
1

For Cutztr/AP, the combustion duration time and flame area of Cutztr/AP_2_ are larger than those of Cutztr/AP_1_ and Cutztr/AP_3_ (Figure S9), indicating that the optimal oxygen-to-fuel ratio is achieved, resulting in full and complete combustion. As shown in Figure S10, the combustion duration of Cutztr@AP_2_ is shorter than that of Cutztr@AP_1_ and Cutztr@AP_3_, while the flame area is larger compared Cutztr@AP_1_ and Cutztr@AP_3_. This difference can be attributed to the close contact between the oxidizer and fuel, facilitating rapid and intense combustion.

### 3.4 Pressure Performance

**Figure 6:**
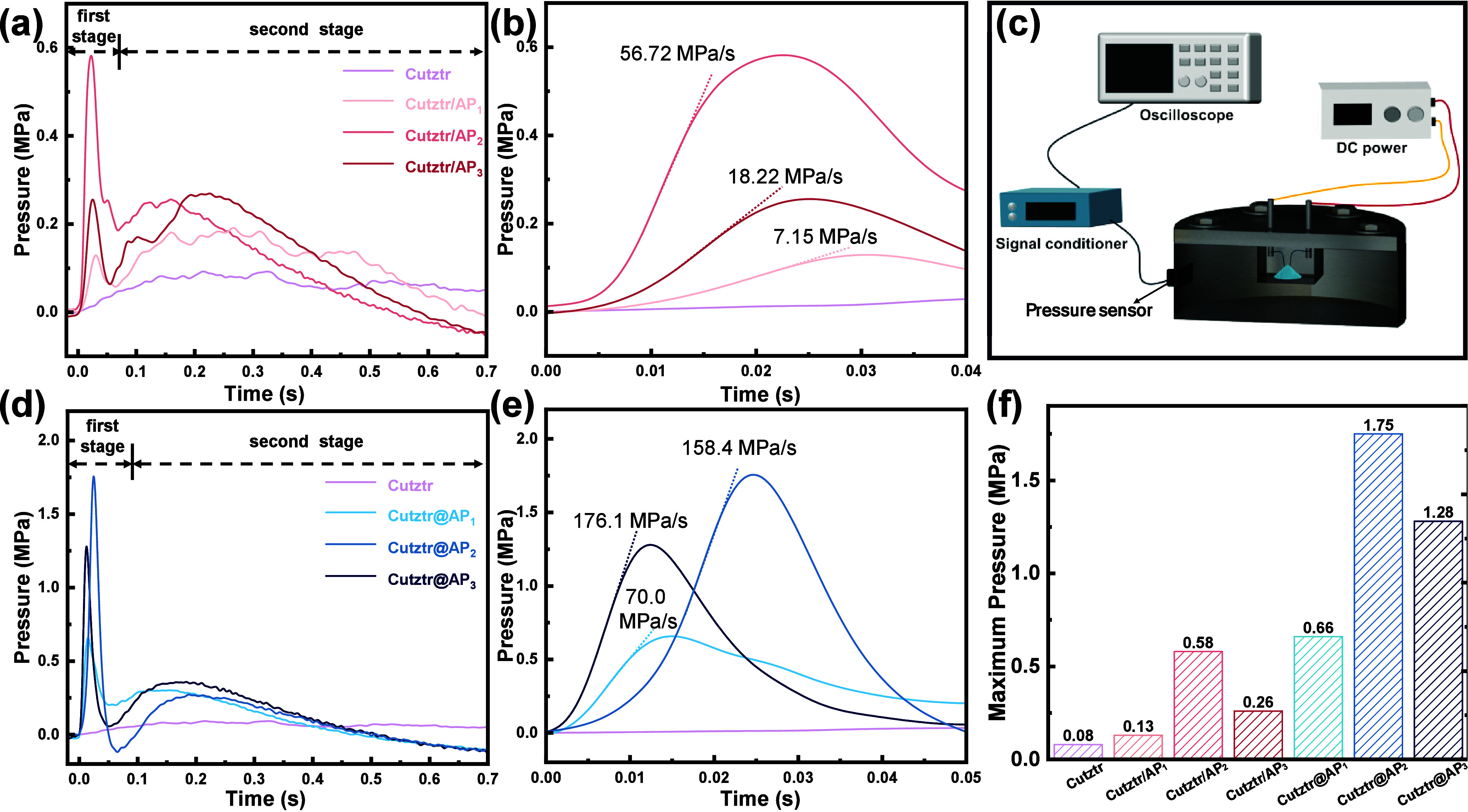
(a and d) Pressure traces vs time of Cutztr, Cutztr/AP, and Cutztr@AP obtained by close-bomb experiments. (b and e) Enlarged pressure–time curves. (c) Schematic of the homemade experimental apparatus of the pressure test. (f) Maximum pressure of the samples.

The dynamic pressure curves of Cutztr, Cutztr/AP, and Cutztr@AP powders were tested by using closed-bomb experiments. In Figure 6, it is evident that all samples can be ignited and generate pressure pulses within a confined environment. Due to poor oxygen balance and incomplete combustion in a relatively confined space, Cutztr does not perform as well as Cutztr/AP and Cutztr@AP in terms of peak pressure, as evidenced by the fact that most samples do not react, as shown in Figure S11.

The peak pressures for Cutztr/AP_1_, Cutztr/AP_2_, and Cutztr/AP_3_ are 0.13, 0.58, and 0.26 MPa, respectively, higher than that of Cutztr, which peaks at approximately 0.1 MPa. The peak pressure of Cutztr/AP exhibits two distinct stages, consistent with the results of the combustion experiments. The first stage occurs from ignition to about 0.05 s, while the second stage ranges from 0.05 to 0.7 s, with the overall reaction time exceeding that of the combustion experiment, possibly due to the increased sample size of approximately 25 mg in this test. Notably, Cutztr/AP_2_ exhibits the highest peak pressure and pressurization rate at 0.58 and 56.72 MPa·s^–1^, respectively, indicating significantly improved reactivity and gas production after ultrasonic mixing of AP and Cutztr. The higher peak pressure in the Cutztr/AP samples correlates with higher pressurization rates, reflecting a positive relationship between the pressure and combustion propagation.

Furthermore, Cutztr@AP synthesized via *in situ* assembly shows a superior gas production performance, possibly due to the closer proximity between the oxidizer and fuel, which increases the reaction rate and reactivity, resulting in more gaseous products. The maximum pressure and pressurization rate of Cutztr@AP_2_ are nearly 3 times those of Cutztr/AP_2_, significantly outperforming Cutztr/AP. The relatively high peak pressure of Cutztr@AP is probably due to the nitrogen-rich ligand and oxygen-rich perchlorate in Cutztr, releasing a significant amount of gas during combustion (e.g., N_2_ and CO_2_, as shown in Figure 4e). Additionally, Cutztr@AP releases a higher heat of reaction, contributing to its higher peak pressure. Among these samples, Cutztr@AP_2_ exhibits the highest peak pressure (1.75 MPa), surpassing those of Cutztr@AP_1_ (0.66 MPa) and Cutztr@AP_3_ (0.28 MPa). Its peak pressure and pressurization rate are higher than that of Al/CuO (*P*_max_: pressure = 1.01 MPa and pressurization rate = 93.3 MPa·s^–1^).^51^

## 4 Conclusions

In summary, a promising *in situ* approach for preparing fuel–oxidant self-assembled Cutztr@AP was demonstrated and compared with ultrasonically mixed Cutztr/AP. The nanoscale size of Cutztr enables its close integration with micrometer-sized AP, facilitating the formation of tightly bound Cutztr@AP energetic composites, thereby reducing the mass-transfer distance between fuels and oxidizers. The thermal behaviors, combustion, and pressure output performances of Cutztr@AP and Cutztr/AP were thoroughly analyzed and investigated. The following conclusions were drawn:

(1) The heat release of Cutztr@AP prepared by *in situ* self-assembly can be further improved to 2378.2 J·g^–1^, which is higher than that of Cutztr/AP_2_ (2000.0 J·g^–1^). In comparison with Cutztr/AP prepared by an ultrasonic mixing method, Cutztr@AP showed a lower initial temperature and stronger reactivity.

(2) Cutztr can effectively react with AP as fuel, resulting in Cutztr@AP exhibiting a faster and more intense combustion process compared to Cutztr. The combustion duration is about one-fifth that of Cutztr, and the flame area is approximately 1.7 times larger.

(3) Importantly, Cutztr@AP possesses superior and rapid pressure output characteristics compared to Cutztr and Cutztr/AP due to improved oxygen balance and more complete reaction resulting from its compact structure.

## Supporting Information Available

FCM algorithm for image recognition and the materials used in the experiment and their specification, EDS results of Cutztr, SEM images of raw AP, Cutztr/AP, and Cutztr@AP, EDS results of Cutztr@AP, high-resolution XPS spectra of Cutztr, Cutztr/AP, and Cutztr@AP for C 1s, TG–DSC results of AP, segmentation results of Cutztr, Cutztr/AP_2_, and Cutztr@AP_2_, sequential images of combustion results of Cutztr/AP and Cutztr@AP, combustion residue of Cutztr, Cutztr/AP_2_, and Cutztr@AP_2_, and a table of TG–DSC parameters (PDF)
