# Supplementary material for: In Situ Assembly of 3-(Tetrazol-5-yl)triazole Complexes with Ammonium Perchlorate for High-Performance Energetic Composites
Source: ACS Appl Mater Interfaces. 2025 Jan 10;17(3):5391–400. doi: 10.1021/acsami.4c20164 (PMC11758780; doi:10.1021/acsami.4c20164)
Supplement: Supplementary file 1 — am4c20164_si_001.pdf [file am4c20164_si_001.pdf]

Supporting Information

In *Situ* Assembly of 3-(Tetrazol-5-yl) Triazole Complex with Ammonium Perchlorate for High-performance Energetic Composites

Ke-Juan Meng [a], Kunyu Xiong [a], Iftikhar Hussain [a], Momang Tian [a], Xinwen Ma [a], Yuxiang Li [a], Qi-Long Yan[b], Kaili Zhang* [a]

[a] Department of Mechanical Engineering, City University of Hong Kong, 83 Tat Chee Avenue, Hong Kong SAR, China

[b] State Key Laboratory on Solid Rocket Propulsion, Northwestern Polytechnical University, Xi'an 710072, China

*Corresponding author: kaizhang@cityu.edu.hk (Prof. Kaili. Zhang)

Contents

S1. General information

S2. Supplementary figures

S3. Supplementary tables

S1. General information

S1.1 FCM algorithm

The idea of the algorithm is that after initializing the cluster center of the image, the membership degree of each pixel to the cluster center and the new cluster center are solved according to the formula, to achieve the optimal target criterion function. The objective function of FCM algorithm is:

$$J(U,V) = \sum_{k=1}^{n} \sum_{i=1}^{c} u_{ik}^{m} d_{ik}^{2}(x_k, v_i) \tag{1}$$

In the **Formula 1**, c represents the number of clusters, $u_{ik}(i=1,\cdots,c,k=1,\cdots,n)$ represents the membership degree of x_k to the ith cluster, $u_{ik} \in [0,1]$, m represents the fuzzy coefficient, and $d_{ik}(x_k, v_i)$ represents the Euclidean distance of x_k to the ith cluster:

$$d_{ik}(x_k, v_i) = \|x_k - v_i\| \tag{2}$$

The Lagrange method is used to obtain:

$$u_{ik} = \left(\sum_{j=1}^{c} \left(\frac{d(x_k, v_i)}{d(x_k, v_j)} \right)^{\frac{2}{m-1}} \right)^{-1} \tag{3}$$

$$v_i = \frac{\sum_{k=1}^{n} (u_{ik})^m x_k}{\sum_{k=1}^{n} (u_{ik})^m} \tag{4}$$

The flow of the algorithm is: (1) initialize, v_i, m and m. (2) Update the membership matrix according to **Formula 3**. (3) Update the clustering center according to **Formula 4**. (4) Iterate until $\|v_{i+1} - v_i\| < \varepsilon(\varepsilon > 0)$, in which ε is the threshold given in advance, and otherwise continue to perform the step.

S2. Supplementary figures

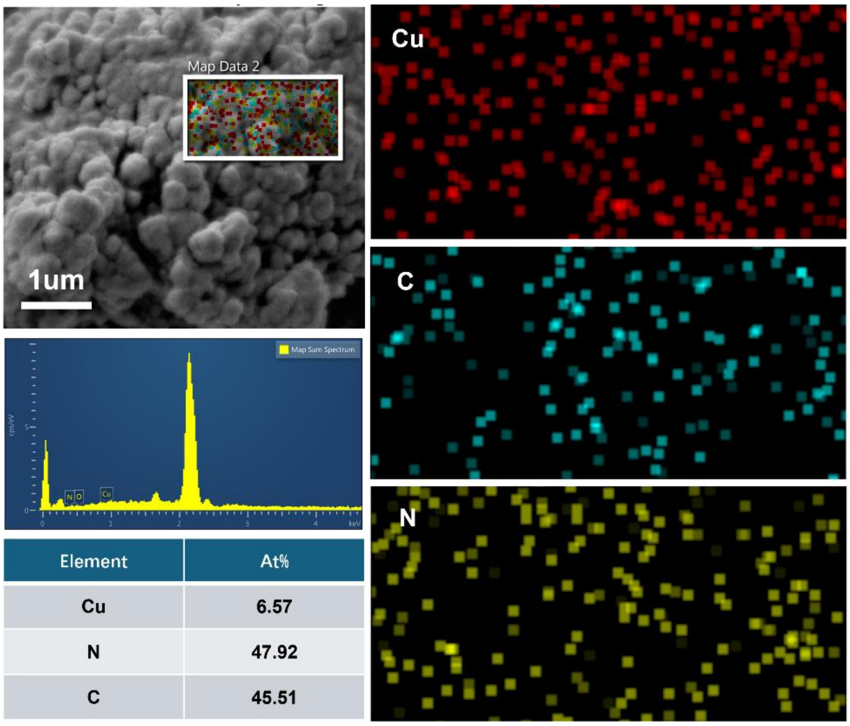

Figure S1. EDS results of Cutztr.

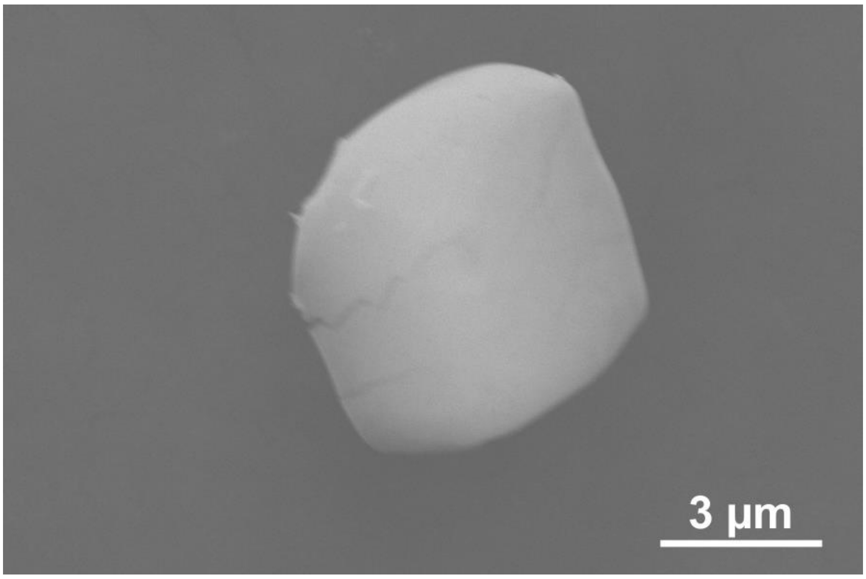

Figure S2. SEM image of raw AP.

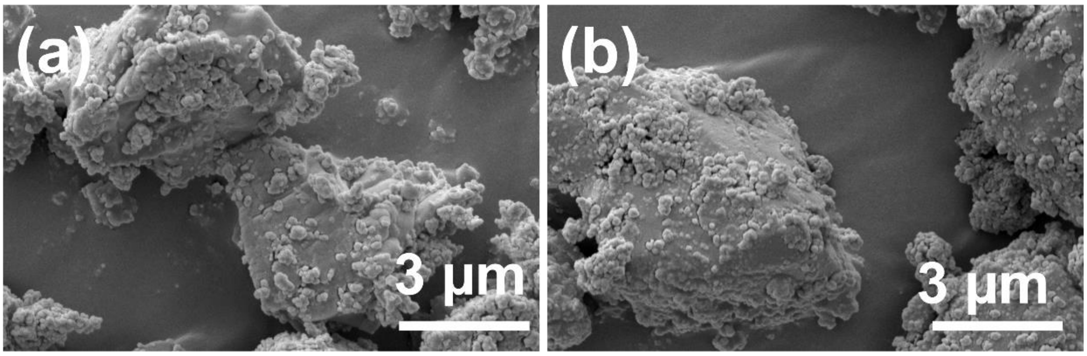

Figure S3. SEM images of Cutztr/AP_1 and Cutztr/AP_3.

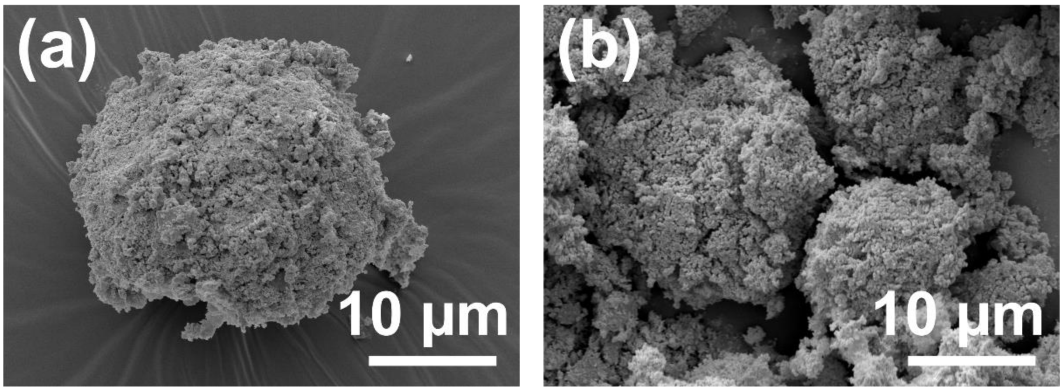

Figure S4. SEM images of Cutztr@AP_1 and Cutztr@AP_3.

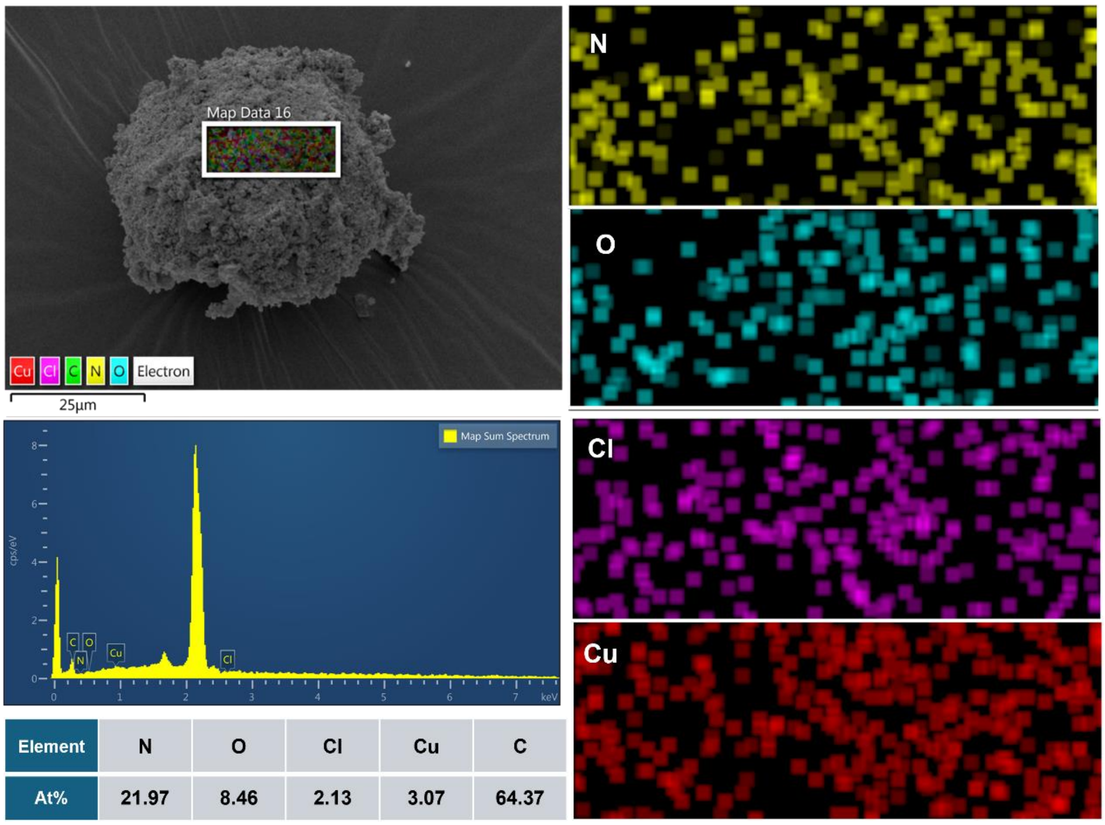

Element	N	O	Cl	Cu	C
At%	21.97	8.46	2.13	3.07	64.37

Figure S5. EDS results of Cutztr@AP.

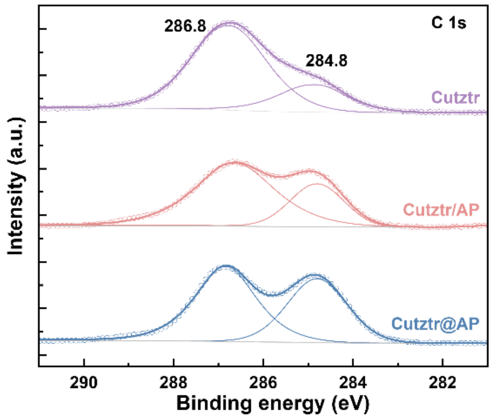

Figure S6. High resolution XPS spectra of Cutztr, Cutztr/AP and Cutztr@AP for C1s.

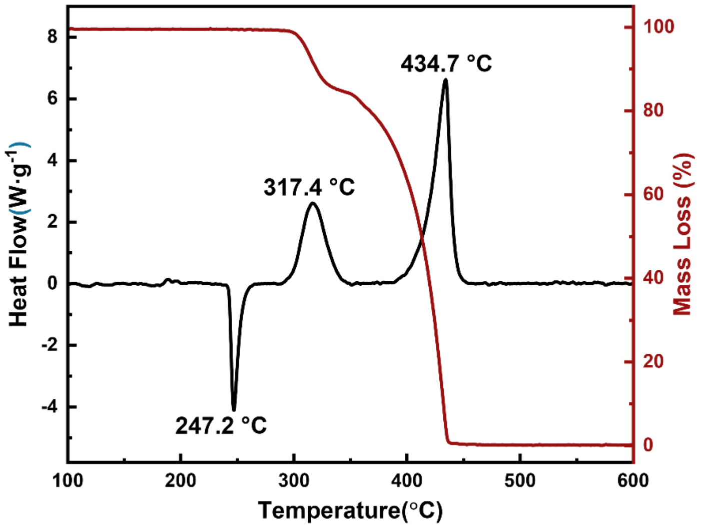

Figure S7. TG-DSC curves of AP.

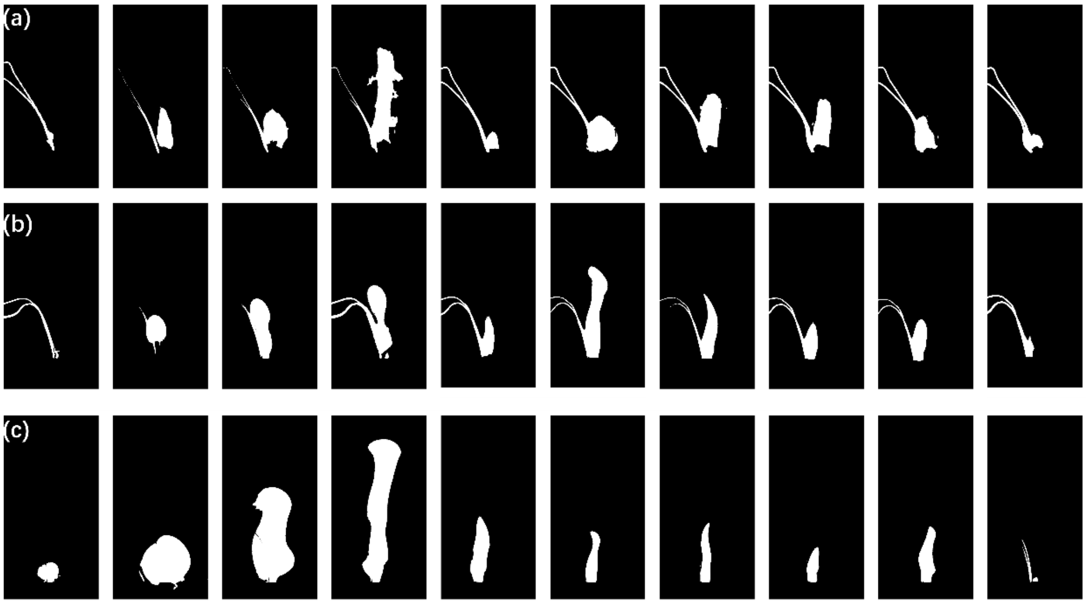

Figure S8. The segmentation results of Cutztr, Cutztr/AP$_2$ and Cutztr@AP$_2$.

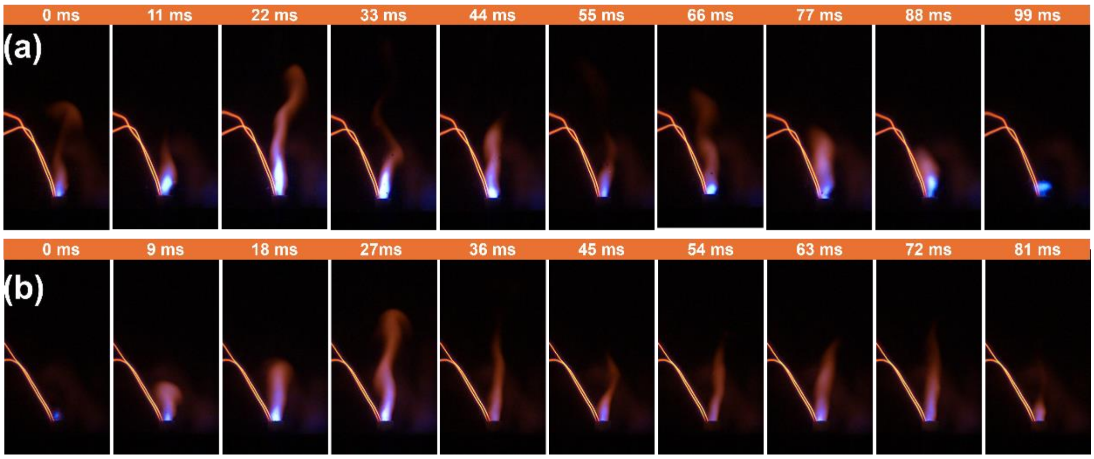

Figure S9. The sequential images of combustion results of (a) Cutztr/AP_1 and (b) Cutztr/AP_3.

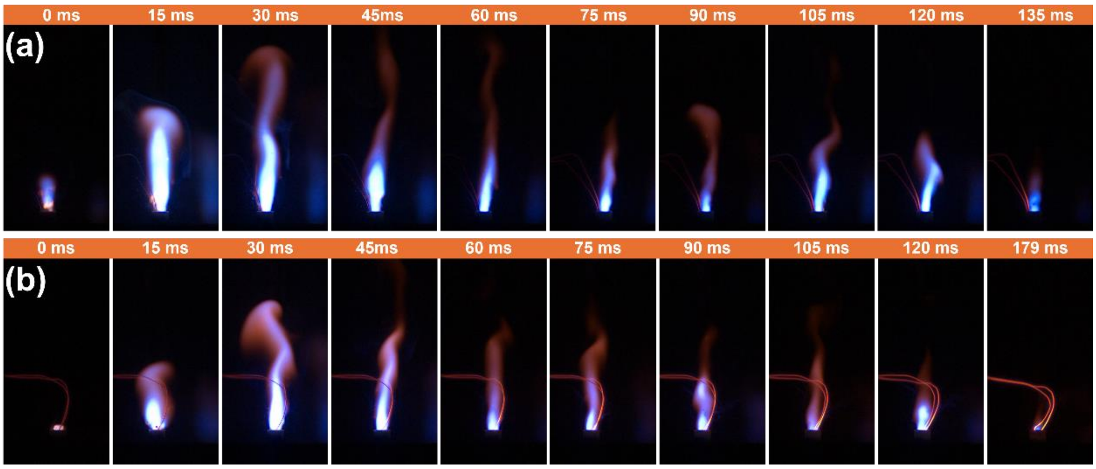

Figure S10. The sequential images of combustion results of (a) Cutztr@AP_1 and (b) Cutztr@AP_3.

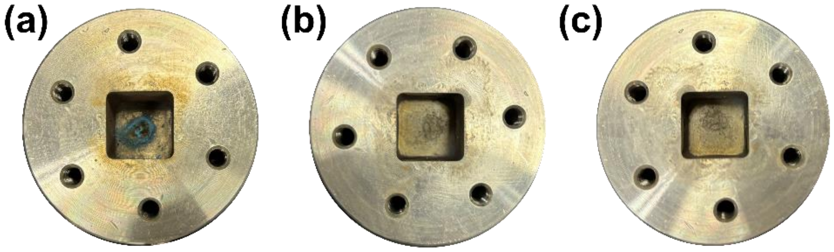

Figure S11. The combustion residue of (a) Cutztr; (b) Cutztr/AP_2; (c) Cutztr@AP_2.

S3. Supplementary tables

Table S1 TG-DSC parameters of samples.

Samples	Atmosphere	DSC					TG
		T_o(°C)	T_p(°C)	T_e(°C)	ΔH($J\cdot g^{-1}$)	$HeatFlow_{max}$(mW/mg)	Re (%)
AP	Ar		317.4 (1st)<br>434.7 (2nd)		476.1	6.8	0.04
Cutztr	Ar	276.2	336.9	392.6	1018.0	4.5	65.7
Cutztr	air	274.8	369.0	399.7	6627.2	81.3	27.3
Cutztr/AP_1	Ar	254.5	337.8	375.8	1881.6	14.8	26.6
Cutztr/AP_2	Ar	252.5	338.2	376.2	2000.0	13.2	21.0
Cutztr/AP_2	air	258.0	337.8	403.7	2137.0	16.6	14.7
Cutztr/AP_3	Ar	256.1	338.8	374.3	1726.0	12.7	18.5
Cutztr@AP_1	Ar	250.5	317.6	357.5	2110.9	12.9	18.9
Cutztr@AP_2	Ar	251.1	323.2	354.2	2378.2	17.9	16.7
Cutztr@AP_2	air	251.8	327.3	392.5	2755.1	24.0	10.8
Cutztr@AP_3	Ar	253.1	325.8	358.5	2332.0	16.1	15.3

a): T_o, onset temperature of the peaks; T_p, peak temperature of exothermic process; T_e, the end temperature for heat change; ΔH, heat release; Re, residual mass.
